# Ultrasound stimulation increases proliferation of MC3T3-E1 preosteoblast-like cells

**DOI:** 10.1186/2050-5736-2-1

**Published:** 2014-01-02

**Authors:** Amit Katiyar, Randall L Duncan, Kausik Sarkar

**Affiliations:** 1 Department of Mechanical Engineering, University of Delaware, Newark, DE 19716, USA; 2 Department of Biological Sciences, University of Delaware, Newark, DE 19716, USA; 3 Department of Mechanical and Aerospace Engineering, George Washington University, Washington, DC 20052, USA

**Keywords:** Osteoblast, Proliferation, Ultrasound, Mechanical stimulation, LIPUS

## Abstract

**Background:**

Mechanical stimulation of bone increases bone mass and fracture healing, at least in part, through increases in proliferation of osteoblasts and osteoprogenitor cells. Researchers have previously performed *in vitro* studies of ultrasound-induced osteoblast proliferation but mostly used fixed ultrasound settings and have reported widely varying and inconclusive results. Here we critically investigated the effects of the excitation parameters of low-intensity pulsed ultrasound (LIPUS) stimulation on proliferation of MC3T3-E1 preosteoblastic cells in monolayer cultures.

**Methods:**

We used a custom-designed ultrasound exposure system to vary the key ultrasound parameters—intensity, frequency and excitation duration. MC3T3-E1 cells were seeded in 12-well cell culture plates. Unless otherwise specified, treated cells, in groups of three, were excited twice for 10 min with an interval of 24 h in between after cell seeding. Proliferation rates of these cells were determined using BrdU and MTS assays 24 h after the last LIPUS excitation. All data are presented as the mean ± standard error. The statistical significance was determined using Student's two-sample two-tailed *t* tests.

**Results:**

Using discrete LIPUS intensities ranging from 1 to 500 mW/cm^2^ (SATA, spatial average-temporal average), we found that approximately 75 mW/cm^2^ produced the greatest increase in osteoblast proliferation. Ultrasound exposures at higher intensity (approximately 465 mW/cm^2^) significantly reduced proliferation in MC3T3-E1 cells, suggesting that high-intensity pulsed ultrasound may increase apoptosis or loss of adhesion in these cells. Variation in LIPUS frequency from 0.5 MHz to 5 MHz indicated that osteoblast proliferation rate was not frequency dependent. We found no difference in the increase in proliferation rate if LIPUS was applied for 30 min/day or 10 min/day, indicating a habituation response.

**Conclusion:**

This study concludes that a short-term stimulation with optimum intensity can enhance proliferation of preosteoblast-like bone cells that plays an important role in bone formation and accelerated fracture healing, also suggesting a possible therapeutic treatment for reduced bone mass.

## Background

Bone fracture healing is a complex physiological process that sequentially involves initial inflammation, soft and hard-callus formation and finally bone repair and remodeling [1]. Every year, millions of fractures are reported worldwide—according to the World Health Organization, in 2000, approximately 9.0 million osteoporotic fractures were reported worldwide, half of them in America and Europe. Even with the state of the art clinical treatments, 5%–10% of bone fractures in the USA fail (nonunion) or take more than usual time (delayed union) to heal [2]. The extended treatment might require surgical intervention for possible bone-grafting and/or internal fixation.

Mechanical forces are required for skeletal homeostasis [3,4]. LIPUS is a nonthermal and nondestructive source of mechanical energy (i.e. intensity = 5–100 mW/cm^2^) [5]. Application of low-intensity pulsed ultrasound (LIPUS) has been approved for treatment of fresh as well as nonunion fractures by the Food and Drug Administration. LIPUS resulted in a significant reduction in the overall fracture healing time in several animal models [6,7] and clinical trials [8-11]. LIPUS can produce micromechanical strains in tissues which, in turn, can trigger several cellular responses [12]. However, they are not completely understood [13-15]. Previous researchers investigated the effects of LIPUS on various cellular activities such as cell proliferation [16], cell differentiation [5], extracellular collagen synthesis, protein and factor synthesis, gene expression, and cytosolic calcium levels [16].

To date, investigations of LIPUS stimulation of bones concentrated on the bone-forming cells, the osteoblast [17,18]. *In vitro* studies of ultrasound-induced osteoblast proliferation, however, have reported widely varying results. Doan et al. [19] found significant but unevenly distributed increase in human mandibular osteoblast cell proliferation (32% at 5 mW/cm^2^ spatial average (SA), 5% at 15 mW/cm^2^ SA, 35% at 30 mW/cm^2^ SA, and 18% at 50 mW/cm^2^ SA) using a near-field continuous ultrasound exposures at 45 kHz. At 1 MHz pulsed ultrasound excitation, increased cell proliferation was observed in the same study but only at relatively higher intensities (47% at 0.7 W/cm^2^ spatial average-pulse average (SAPA) and 37% at 1 W/cm^2^ SAPA). Hayton et al. [20] found approximately 10% rise of proliferation in human osteoblast-like cells Saos-2 due to a 40-min excitation of standard LIPUS exposure—1.5 MHz frequency, 1 kHz PRF (pulse repetition frequency), 200 μs pulse duration and 30 mW/cm^2^ intensity. In contrast, Suzuki et al. [5,21] showed that there is no effect on cell proliferation for a near-field and 20-min standard LIPUS exposure to rat osteoblast-like cells ROS 17/2.8. Most recently, Kang et al. [22] studied the effects of 20 min a day stimulation by a low-intensity ultrasound (1 MHz, 30 mW/cm2 continuous sine wave) in combination with cyclic vibratory strain (1 Hz, 10% strain) on MC3T3-E1 cells in a 3D scaffold. The stimulation did not change the cell proliferation over a period of 10 days, but significantly up-regulated several gene expressions—COL-I, OC, RUNX2, and OSX—indicating accelerated differentiation.

It is clear that LIPUS parameters for peak proliferation vary and the effects on osteoblast or osteoblast-like cells are not always the same. There is a need for a systematic study of the LIPUS effects varying the parameters of excitation such as intensity, frequency, and waveform. The objective of this study was to determine the effects of near-field LIPUS-induced mechanical stimulation on osteoblast cell proliferation in a monolayer culture and to understand its dependence on key ultrasound parameters: intensity, frequency, and the excitation period.

## Methods

### Cell culture

The MC3T3-E1 cells (passages 20–27), a preosteoblastic cell line, were cultured in α-minimal essential medium (Sigma Chemical, St. Louis, MO, USA) containing 10% fetal bovine serum (Gibco, New York, NY, USA), 100 units/ml penicillin G (Sigma) and 100 μg/ml streptomycin (Sigma). Cells were cultured in a humidified incubator at 37°C with 95% air and 5% CO_2_ and subcultured every 72 h.

### Ultrasound excitation

**Figure 1:**
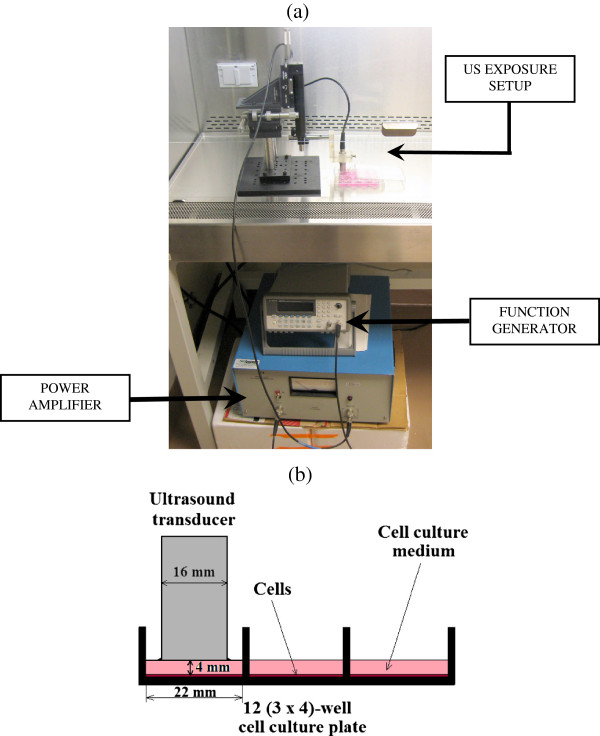
**Setup. (a)** Customized ultrasound exposure system. **(b)** Schematic representation of ultrasound exposure setup.

In previous studies, modified clinical devices have been used to produce LIPUS [16,18,19,23,24]. However, to obtain better control on the characteristic parameters of US, we used a custom-designed ultrasound exposure system. The arrangement of electronic instruments for ultrasound exposure is shown in Figure 1a. A programmable function generator (33250A, Agilent, Palo Alto, CA, USA) produced standard 200 μs long pulses (sinusoidal waves) at 1 kHz PRF. The transmit signal was amplified by a broadband 55 dB laboratory RF power amplifier (model A-150; ENI, Rochester, NY, USA) and then supplied to a single-element unfocused immersion transducer (part number A306S, GE Panametrics, Waltham, MA, USA). The transducer had an outside diameter of 16 mm and a center frequency of 2.5 MHz. For frequency variation study, we used transducers with different center frequencies.

The ultrasound transducer and an XYZ positioning stage (Newport Corp., CA, USA) were sterilized with 75% of ethanol and kept under ultraviolet light for at least 2 h before the experiments. Based on the diameter of the transducer (head area 2.01 cm^2^), we found 12-well cell culture plates (growth area 3.80 cm^2^) appropriate for our experiments. MC3T3-E1 cells were seeded on the bottom of the 12-well plate with 1.5 mL of cell culture medium. The transducer head was positioned vertically over the culture well, just touching the surface of the medium (Figure 1b). In this configuration, the distance between the transducer head and the bottom of the dish was approximately 4 ± 0.5 mm (determined from the XYZ positioning as well as the cross-section area of the cell and the volume of the medium) and kept constant for all the experiments. Note that the cells are in the near field, as in several other investigations [19,21] and, therefore, are subjected to a spatially nonuniform field. However, the setup has the advantage of direct stimulation by the immersed transducer unimpeded by an intervening medium which would otherwise attenuate the signal. Note that several animal and clinical trials of therapeutic ultrasound involved near-field stimulation by transducers in direct contact with the skin [8,9,11]. Li et al. [24] specifically determined the optimum intensity for far-field stimulations and found it to be comparable to the near-field values quoted in the literature. For the stimulation used here, the proper spatial averages are computed using the relations described in the Appendix.

MC3T3-E1 cells at 50%–60% confluence (2.28 × 10^4^ cells per well ≈ 6 × 10^3^ cells/cm^2^) were seeded in 12-well cell culture plates. We experimentally verified that these cells remain in linear phase of proliferation up to approximately 4 × 10^4^ cells per well. Cells received their first US stimulation 24 h after seeding. Unless otherwise mentioned, stimulation was given twice for 10 min and at an interval of 24 h. The control group underwent the same experimental treatment with the ultrasound powered off.

We note that there is a possibility of indirect transfer of mechanical energy of ultrasound to the neighboring wells [25]. We investigated the secondary ultrasound stimulation in neighboring wells and found it less than 1% to that transferred directly. We note that our setup for ultrasound stimulation has been frequently used for studying its cellular effects and release properties of drug bearing vesicles. Recent studies have indicated that in this setup, reflections from the air-water interface create a standing wave pattern giving rise to a spatially varying acoustic field [20,21]. However, note that unlike in a suspension of drug bearing particles, ours is a monolayer of cells with dimension which is much smaller than the wavelength. Therefore, the variation of excitation between cells is negligibly small, and the current setup is adequate for our purpose.

### Determination of cell proliferation

#### BrdU assay

The BrdU ELISA (Amersham Cell Proliferation Biotrak ELISA system, version 2, GE Healthcare Bio-Sciences Corp., Piscataway, NJ, USA) is based on incorporation of 5-bromo-2′-deoxyuridine (BrdU) during DNA synthesis in proliferating cells. To quantify the cell proliferation (24 h after second US stimulation), BrdU labeling reagent diluted with cell culture medium (0.4 ml of 1:1,000 *v*/*v*) was added to each well of 12-well plate and the cells were reincubated for 2 h in a humidified incubator at 37°C with 95% air and 5% CO_2_. During the labeling period, BrdU is incorporated in place of thymidine into the DNA of proliferating cells. The BrdU labeling reagent was then removed from the well, and 0.4 mL of fixative solution (for cell fixation and DNA denaturation) supplied in the kit was added to each well, and the cells were incubated for an additional 30 min at room temperature (RT). The denaturation of the DNA is necessary to improve the accessibility of the incorporated BrdU for detection by the antibody. The fixative solution was then removed and 0.4 mL of 1:10 diluted blocking buffer (also supplied in the kit to block the remaining binding surface and prevent any nonspecific binding of the antibodies) was added to each well. Following incubation at room temperature for 30 min, the blocking buffer was removed and 0.4 mL of 1:100 of diluted peroxidase-labeled anti-BrdU (monoclonal antibody from mouse cells conjugated to peroxidase, lyophilized, and stabilized) working solution was added. The peroxidase-labeled anti-BrdU solution is diluted with supplied antibody dilution solution. Cells were incubated in this solution at room temperature for 90 min. The peroxidase-labeled anti-BrdU binds to the BrdU, which is incorporated in newly synthesized cellular DNA. The anti-BrdU working solution was then removed, and the cells were washed with 1 ml of 1:10 diluted wash buffer solution (phosphate buffer saline (PBS), 10× concentrate) three times at room temperature. Room temperature-equilibrated 3,3′5,5-tetramethylbenzidine (TMB) substrate solution (0.4 mL) in 15% (*v*/*v*) dimethyl sulfoxide (DMSO) was then added to each well. The immune complex formed after adding the peroxidase-labeled anti-BrdU reacts with TMB substrate. After approximately 10 min, a light blue color solution is obtained and the reaction was then stopped by adding 100 μL of 2 M H_2_SO_4_ solution to each well. The optical density (absorbance) of 150 μL of resultant yellowish color solution was read at 450 nm in a 96-well microplate spectrophotometer. The absorbance values correlate directly to the amount of DNA synthesis and thereby to the number of proliferating cells in culture.

#### MTS assay

To corroborate the BrdU data, osteoblast cell number was also determined using [3-(4,5-dimethylthiazol-2-yl)-5-(3-caroxymethoxyphenyl)-2-(4-sulfophenyl)-2H-tetrazolium (MTS) assay (CellTiter 96 Aqueous, Promega, Madison, WI, USA). This assay is colorimetric based on the reduction of the MTS tetrazolium by the living cells to a formazan product. The absorbance of the formazan product is measured at 490 nm and the generation of this product is directly proportional to the cell mass. In this assay, 80 μL of the MTS solution was diluted into 0.4 ml of cell culture medium and added to each well. The cell culture plate was incubated 37°C for 2 h in a humidified 5% CO_2_ atmosphere. The absorbance was recorded at 490 nm using a 96-well plate reader.

In this study, each single experiment was repeated at least three times on three different passages of MC3T3-E1. All data are presented as the mean ± standard error (SE). The statistical significance was determined using Student's two-sample two-tailed *t* tests. Values of *p* <0.05 were considered to be statistically significant.

## Results

MC3T3-E1 osteoblasts responded to LIPUS with increase in cell proliferation, and the details are provided in the following subsections.

### Intensity dependence of osteoblast proliferation

**Figure 2:**
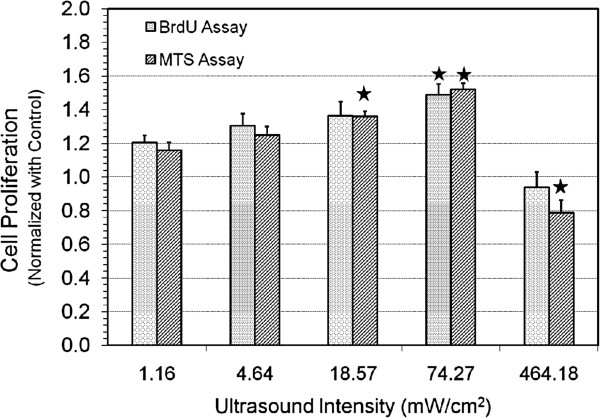
**Change in normalized proliferation of MC3T3-E1 cells with ultrasound intensity.** Change in proliferation of ultrasound stimulated MC3T3-E1 cells (normalized with control) at different ultrasound intensities (SATA) with frequency = 1.5 MHz, PRF = 1 kHz, pulse duration = 200 μs and exposure time = 10 min. Values significantly different from control group have been indicated by *filled stars* for *p* < 0.05.

To determine the peak proliferative response, the ultrasound intensity was first varied over the range of 1 to 500 mW/cm^2^ (SATA). The exposure time was set at 10 min and all other parameters (frequency = 1.5 MHz, PRF = 1 kHz, pulse duration = 200 μs) were kept the same. We varied the input electrical signal to transducer by a factor of 2 and 2.5, which increased the ultrasound intensity by a factor of 4 and 6.25 respectively (1.16, 4.64, 18.57, 74.27, and 464.18 mW/cm^2^). Figure 2 shows the effect of ultrasound excitation at different intensities on osteoblast cell proliferation. The BrdU assay shows that the cell proliferation increased approximately 20%, 30%, 36%, and 49% for the four lower intensities, respectively. At the higher intensity of approximately 465 mW/cm^2^, the proliferation decreased by approximately 6%, showing an inhibitory effect on osteoblast cell growth. Some cells were also found detached from the base of the cell culture plate after the excitation at this higher intensity.

The BrdU measurements were validated with an MTS assay. As shown in Figure 2, the increase in cell proliferation was approximately 16%, 25%, 36%, and 52% for the four lower intensities respectively. Further, stimulation at the higher intensity of approximately 465 mW/cm^2^ was detrimental to cell viability with a proliferation decrease of approximately 21%. These results obtained by an independent assay are similar to those obtained by the BrdU assay.

**Figure 3:**
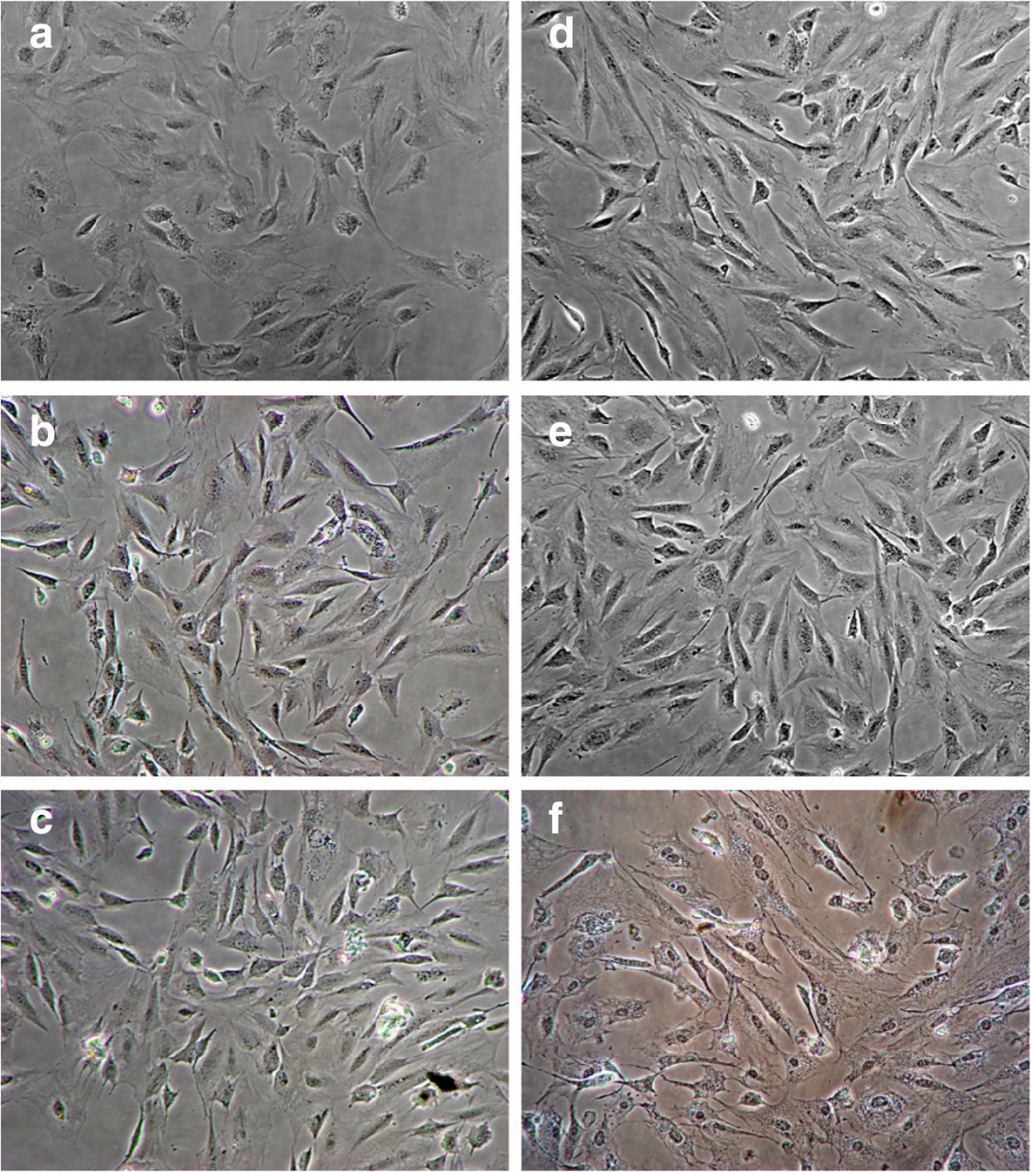
**Microscopic images of MC3T3-E1 cells at the end of LIPUS stimulation.** Microscopic images of cell growth after two 10-min ultrasound excitations at 24-h interval and at following ultrasound intensities **(a)** control, **(b)** 1.16 mW/cm^2^, **(c)** 4.64 mW/cm^2^, **(d)** 18.57 mW/cm^2^, **(e)** 74.27 mW/cm^2^, and **(f)** 464.18 mW/cm^2^. Magnification × 20.

Microscopic images of MC3T3-E1 preosteoblastic cells after two 10-min US excitations at 24-h interval and at different ultrasound intensities are shown in Figure 3. These images were taken at the central portion of the respective wells where maximum ultrasound intensity was delivered. Though the increase in cell number due to US stimulation over control group is not always visually distinct, Figure 3 shows that the cell count increased approximately 19%, 32%, 39%, and 53% for the four lower intensities, respectively. For the higher LIPUS intensity of approximately 465 mW/cm^2^, cells distinctly look compressed and damaged.

### Frequency dependence of proliferation in MC3T3-E1 cells

**Figure 4:**
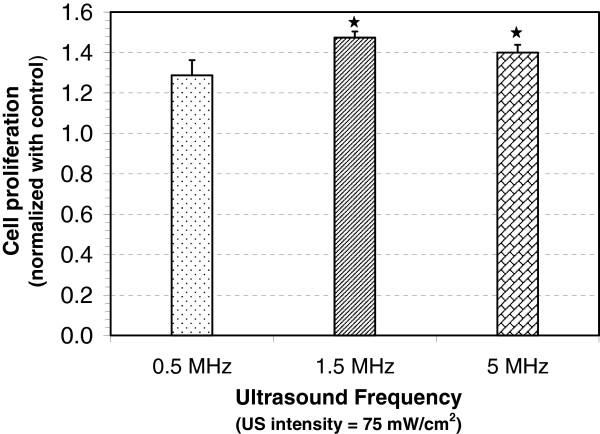
**Change in proliferation of MC3T3-E1 cells with ultrasound frequency.** Change in proliferation of ultrasound stimulated MC3T3-E1 cells (normalized with control) at excitation frequencies of 0.5, 1.5, and 5 MHz with US intensity = 75 mW/cm^2^ (SATA), PRF = 1 kHz, pulse duration = 200 μs, and exposure time = 10 min. Values that are significantly different from control group have been indicated by for *p* < 0.05.

Once the optimum intensity was identified, effects of excitation frequency were investigated over frequencies ranging from 0.5 to 5 MHz at the optimal intensity (75 mW/cm^2^) with a 10-min exposure time. In this experiment, we ensured that the pulse duration (200 μs) and PRF (1 kHz) remained the same by changing the number of cycles while changing the frequency. Figure 4 shows that the ultrasound stimulation increased osteoblast cell proliferation at all three frequencies. However, there is no statistically significant difference in proliferation at different frequencies.

### Optimum ultrasound excitation period for peak proliferative response

**Figure 5:**
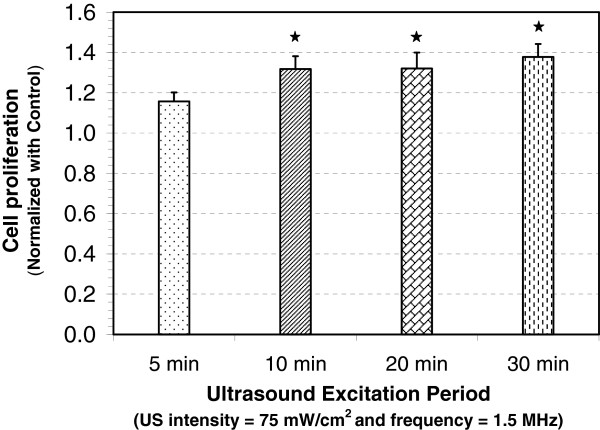
**Change in proliferation of MC3T3-E1 cells ultrasound exposure period.** Change in proliferation of ultrasound-stimulated MC3T3-E1 cells (normalized with control) at different ultrasound exposure periods of 5, 10, 20, and 30 min with ultrasound intensity = 75 mW/cm^2^ (SATA), frequency = 1.5 MHz, PRF = 1 kHz, and pulse duration = 200 μs. Values significantly different from control group have been indicated by *filled star* for *p* < 0.05.

In several previous *in vitro* studies, researchers have explored the effects of ultrasound application from a few seconds to several hours [16,20,26,27]. To determine if osteoblast proliferation was dependent on the excitation period, we varied it for 5, 10, 20, and 30 min. Figure 5 shows that the ultrasound stimulation increased cell proliferation for each excitation period tested. However, only 10 min and more exposure periods show statistically significant increase over the control group. There was no statistically significant difference among the LIPUS excited groups at different exposure times of 10, 20, and 30 min.

## Discussion

Mechanical stimuli play an important role in the development and maintenance of healthy skeleton. Increased mechanical loading on bone enhances bone formation and suppresses bone resorption to increase bone mass [28,29]. Bone cells sense the mechanical forces and produce biochemical signals to bring the changes in their microenvironment. For example, mechanical loading generates microstrains and causes fluid flow through lacunar and canalicular spaces of the bone. The resulting fluid shear stress can stimulate osteoblast proliferation [30], contributing to the increase in bone mass. Ultrasound is a source of noninvasive mechanical stimulation that can induce acoustic streaming (unidirectional movement in an ultrasonic pressure field), acoustic microstreaming (rapidly rotating small-scale fluid motion around oscillating bubbles), and cavitation (formation of tiny gas bubbles in the tissues as the result of ultrasound vibration) [31]. Because of the low intensity and thereby low mechanical index (0.078 for the intensity of 75 mW/cm^2^ and 0.488 at 465 mW/cm^2^) of the stimulation used, we do not expect any cavitation here [15]. Although we did not try to detect cavitation directly in the setup, the excitation in the range of intensities used here did not generate cavitation in water. Utmost care has been taken to avoid the formation of bubbles in the medium. In any event, different mechanical effects caused by the ultrasound further cause fluid flow in the extracellular space [12] and result in deformation and strain to osteoblasts. Thus, osteoblasts should respond to LIPUS in part due to the same mechanisms that are present in case of shear forces from fluid flow.

Using BrdU and MTS assays, we found enhanced proliferation at different LIPUS intensities with maximum effect at approximately 75 mW/cm^2^. This optimum LIPUS intensity is of the same order reported previously in literature. Reher et al. [23] found the optimum intensity to be 100 mW/cm^2^ SAPA for osteoblastic cell lines with a 200-μs pulse at 1 MHz frequency, whereas intensities higher than approximately 750 mW/cm^2^ led to the inhibition of collagen and noncollagenous proteins. Li et al. [24] found an optimum intensity of 600 mW/cm^2^ SATP or 120 mW/cm^2^ (SATA) for osteoblast growth at 100 Hz PRF, 1:4 duty cycle (2 ms burst period), 1 MHz US frequency, 15 min exposure time, and 24 cm exposure distance. We also found that the US exposure at higher intensities (approximately 465 mW/cm^2^ SAPA) proved detrimental to osteoblasts. In a far-field LIPUS exposure study, Li et al. [24] also reported complete inhibition of cell proliferation at 480 mW/cm^2^ SAPA. High-intensity US exposures have been shown to suppress bone formation in animal models as well [32].

In an attempt to determine the optimum stimulation frequency, we investigated three different frequencies: 0.5, 1.5, and 5 MHz but found no statistically significant difference in osteoblast cell proliferation between them. We also examined the duration of US stimulation to yield peak cellular proliferation. At the optimum US intensity, we found that longer stimulation of 30 min a day was not significantly different from a shorter stimulation of 10 min a day, indicating a habituation response. It has been shown that bone mass increases if loading is applied in intermittent bouts, as bone and osteoblasts become less sensitive to longer mechanical stimulation [33].

How ultrasound alters cell function remains uncertain. Studies using rat bone marrow stromal cells, primary osteoblasts, or intact bone have shown that differentiation markers are increased with 2–30 mW/cm^2^ LIPUS and that this increase corresponds to the increases in focal adhesion kinase (FAK), β-catenin activation, and MAP kinases [34-37]. When the α_5_β_1_ integrins were blocked in primary osteoblasts, LIPUS failed to increase PI3 kinase and β-catenin activity, suggesting that integrins could be the primary sensing molecule for LIPUS [37]. However, several studies in mechanotransduction in bone suggest that other signaling pathways could be sensitive to LIPUS to increase proliferation. Release of ATP and the resultant purinergic signaling increases proliferation, initiates differentiation, and can induce cell death in numerous cells types [38]. We have shown that ATP is released from osteoblasts in response to fluid shear [39] and cyclic hydrostatic pressure [40] and that activation of purinergic receptors is required for mechanically induced bone formation [41]. This release of ATP is mediated through activation of mechanosensitive and voltage-sensitive calcium channels in osteoblasts [39] that could also be responsive to LIPUS. For osteoblast cells, intracellular and extracellular calcium stores and their transport between these stores can play an important role in their response to mechanical stimuli such as LIPUS. Voltage-sensitive calcium channels (VSCCs) have been reported to be the key regulators of intracellular calcium signaling in osteoblasts [39]. In future studies, we plan to investigate possible roles of ultrasound-induced calcium transport in enhanced osteoblast cell proliferation.

## Conclusions

This study demonstrated that the application of near-field LIPUS stimulation is a viable method to enhance osteoblast cell proliferation in monolayer culture. It also supports the possibility that US-induced increased osteoblast proliferation plays an important role in bone formation and accelerated fracture healing. Our findings indicate the need to better define the optimum range of key ultrasound parameters for the maximum stimulation in clinical applications. We have also suggested potential mechanisms of ultrasound-mediated enhancement of osteoblast proliferation to be investigated in future research.

## Appendix

### Intensity measures of pulsed ultrasound

**Figure 6:**
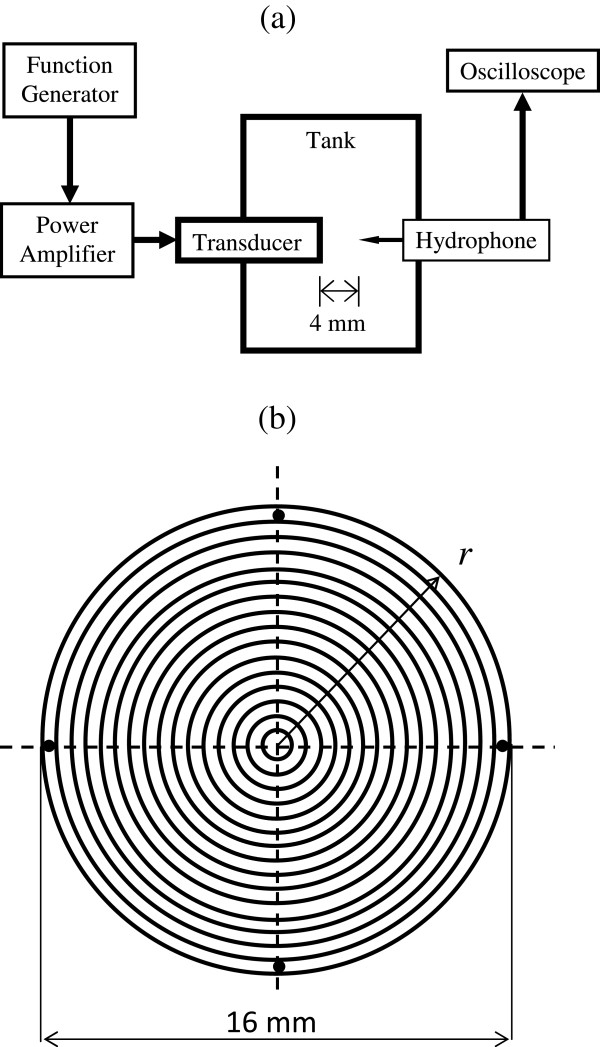
**Setup to measure ultrasound intensity. (a)** Experimental setup for ultrasound intensity measurement using hydrophone. **(b)** Schematic representation of the concentric annular regions and the innermost circular region in ultrasound beam for intensity measurement.

We used an experimental setup shown in Figure 6a for measuring ultrasound intensities. The acoustic pressure was measured through the voltage signal, *V* received by a 0.4-mm needle hydrophone (PZT-Z44-0400, Onda Corporation, CA, USA), and its free-field voltage sensitivity, *M*, as following:

(1) $$\text{pressure},p\left(\mathrm{MPa}\right)=1,000\times \frac{V\left(\mathrm{mV}\right)}{M\left(\frac{\mathrm{\mu V}}{\mathrm{Pa}}\right)}.$$

The plane progressive wave approximation is assumed, so intensity is taken to be proportional to the square of the acoustic pressure. The temporal peak intensity, *I*_TP_(***r***), is related to maximum absolute pressure, *p*_*m*_(***r***), in the medium by the following expression:

(2) $$I_{\mathrm{TP}}\left(r\right)=\frac{P_{m}{\left(r\right)}^{2}}{\mathrm{\rho c}}$$

where ***r*** is the radial coordinate vector on focal surface *S*, *ρ* is the medium density, and *c* is the velocity of the sound in medium. *p*_m_ is half of the measured peak-to-peak pressure at any position ***r***. The pulse average ultrasound intensity, *I*_*PA*_(***r***) and the temporal-average ultrasound intensity, *I*_*TA*_(***r***) were obtained as the following:

(3) $$I_{\mathrm{PA}}\left(r\right)=\frac{1}{T_{P}}\int_{0}^{T_{p}}\frac{p{\left(r,t\right)}^{2}}{\mathrm{\rho c}}\mathrm{dt},$$

(4) $$I_{\mathrm{TA}}\left(r\right)=I_{\mathrm{PA}}\left(r\right)\;\text{duty}\;\text{cycle},$$

where duty cycle is the ratio of pulse duration *T*_*P*_ and the pulse repetition period (inverse of pulse repetition frequency). For a sinusoidal input signal used in this work, *I*_*PA*_(***r***) reduces to

(5) $$I_{\mathrm{PA}}\left(r\right)=\frac{P_{\mathrm{rms}}{\left(r\right)}^{2}}{\mathrm{\rho c}}=\frac{P_{m}{\left(r\right)}^{2}}{2\mathrm{\rho c}}.$$

The spatial-average temporal-average ultrasound intensity (*I*_*SATA*_) and the spatial-average temporal-peak ultrasound intensity were obtained by averaging *I*_*TA*_(***r***) and *I*_*TP*_(***r***) respectively over the beam are as follows [42]:

(6) $$I_{\mathrm{SATA}}=\frac{1}{A_{26}}\int_{S_{26}}I_{\mathrm{TA}}\left(r\right)\mathrm{dS},$$

and

(7) $$I_{\mathrm{SATP}}=\frac{1}{A_{26}}\int_{S_{26}}I_{\mathrm{TP}}\left(r\right)\mathrm{dS}.$$

where *S*_26_ represents integration over the surface where the intensity is greater than 0.25% (-26 dB) of the spatial peak intensity on *S*; and *A*_26_ is the area of surface *S*_26_ (i.e., *A*_26_ is the -26 dB beam area). The -26-dB figure is somewhat arbitrary; it is chosen to encompass essentially the entire ultrasound beam in the integration, yet remain above the hydrophone noise level. The spatial averaging for experimental measurement was performed in discrete manner for which the ultrasound beam was assumed to be composed of several concentric annular areas and the innermost circular area as shown in Figure 6b. On each annular area, four intensity measurements were obtained at points spacing 90° from each other as shown in Figure 6b and were averaged to represent the intensity in that annular area. Only one intensity value was measured at the innermost circular area. Thus, the integration in Equation 6 was computed as

(8) $$I_{\mathrm{SATA}}=\frac{1}{A_{\mathrm{Transducer}}}\sum_{i}I_{{\mathrm{TA}}_{i}}A_{i},$$

where *A*_*i*_ is the area of one of the discretized annular regions or the innermost circular region, *I*_*TAi*_ is the respective average intensity, and *A*_*Transducer*_ = ∑ _*i*_*A*_*i*_ is the area of the transducer face. Here we performed the spatial average over the transducer face area. The spatial average-pulse average intensity is defined as

(9) $$I_{\mathrm{SAPA}}=\frac{I_{\mathrm{SATA}}}{\mathrm{duty}\;\mathrm{cycle}}.$$

## Competing interests

The authors declare that they have no competing interests.

## Authors’ contributions

AK designed the experiments, assembled the ultrasound exposure setup, developed the experimental protocols, conducted the experiments, collected, analyzed and interpreted the data, and drafted the manuscript. RLD and KS conceptualized the idea, supervised the research, analyzed and interpreted the data, and contributed to manuscript writing. All authors read and approved the final manuscript.

## References

[B1] Khan Y Laurencin CT Fracture repair with ultrasound: clinical and cell-based evaluation J Bone Joint Surg Am 2008 90A 138 44 18292369 10.2106/JBJS.G.01218

[B2] Bishop GB Einhorn TA Current and future clinical applications of bone morphogenetic proteins in orthopaedic trauma surgery Int Orthop 2007 31 6 721 7 10.1007/s00264-007-0424-8 17668207 PMC2266667

[B3] Frost HM Wolff law and bones structural adaptations to mechanical usage - an overview for clinician Angle Orthod 1994 64 3 175 88 8060014 10.1043/0003-3219(1994)064<0175:WLABSA>2.0.CO;2

[B4] Woo SLY Kuei SC Amiel D Gomez MA Hayes WC White FC The effect of prolonged physical-training on the properties of long-bone - a study of Wolffs law J Bone Joint Surg Am 1981 63 5 780 7 7240300

[B5] Takayama T Suzuki N Ikeda K Shimada T Suzuki A Maeno M Akeson WH Low-intensity pulsed ultrasound stimulates osteogenic differentiation in ROS 17/2.8 cells Life Sci 2007 80 10 965 71 10.1016/j.lfs.2006.11.037 17174343

[B6] Heybeli N Yesildag A Oyar O Gulsoy UK Tekinsoy MA Mumcu EF Diagnostic ultrasound treatment increases the bone fracture-healing rate in an internally fixed rat femoral osteotomy model J Ultrasound Med 2002 21 12 1357 63 12494977 10.7863/jum.2002.21.12.1357

[B7] Warden SJ Fuchs RK Kessler CK Avin KG Cardinal RE Stewart RL Ultrasound produced by a conventional therapeutic ultrasound unit accelerates fracture repair Phys Ther 2006 86 8 1118 27 16879045

[B8] Mayr E Frankel V Ruter A Ultrasound - an alternative healing method for nonunions Arch Orthop Trauma Surg 2000 120 1–2 1 8 10653095 10.1007/pl00021234

[B9] Heckman JD Ryaby JP Mccabe J Frey JJ Kilcoyne RF Acceleration of tibial fracture-healing by noninvasive, low-intensity pulsed ultrasound J Bone Joint Surg Am 1994 76A 1 26 34 8288661 10.2106/00004623-199401000-00004

[B10] Lubbert PHW van der Rijt RHH Hoorntje LE van der Werken C Low-intensity pulsed ultrasound (LIPUS) in fresh clavicle fractures: a multi-centre double blind randomised controlled trial Inj-Int J Care Injured 2008 39 12 1444 52 10.1016/j.injury.2008.04.004 18656872

[B11] Gebauer D Mayr E Orthner E Ryaby JP Low-intensity pulsed ultrasound: effects on nonunions Ultrasound Med Biol 2005 31 10 1391 402 10.1016/j.ultrasmedbio.2005.06.002 16223643

[B12] Baker KG Robertson VJ Duck FA A review of therapeutic ultrasound: biophysical effects Phys Ther 2001 81 7 1351 8 11444998

[B13] Rubin C Bolander M Ryaby JP Hadjiargyrou M The use of low-intensity ultrasound to accelerate the healing of fractures J Bone Joint Surg Am 2001 83A 2 259 70 11216689 10.2106/00004623-200102000-00015

[B14] Claes L Willie B The enhancement of bone regeneration by ultrasound Prog Biophys Mol Biol 2007 93 1–3 384 98 16934857 10.1016/j.pbiomolbio.2006.07.021

[B15] Pounder NM Harrison AJ Low intensity pulsed ultrasound for fracture healing: a review of the clinical evidence and the associated biological mechanism of action Ultrasonics 2008 48 4 330 8 10.1016/j.ultras.2008.02.005 18486959

[B16] Li JKJ Lin JCA Liu HC Sun JS Ruaan RC Shih C Chang WH Comparison of ultrasound and electromagnetic field effects on osteoblast growth Ultrasound Med Biol 2006 32 5 769 75 10.1016/j.ultrasmedbio.2006.01.017 16677936

[B17] Kokubu T Matsui N Fujioka H Tsunoda M Mizuno K Low intensity pulsed ultrasound exposure increases prostaglandin E-2 production via the induction of cyclooxygenase-2 mRNA in mouse osteoblasts Biochem Biophys Res Commun 1999 256 2 284 7 10.1006/bbrc.1999.0318 10079177

[B18] Ito M Azuma Y Ohta T Komoriya K Effects of ultrasound and 1,25-dihydroxyvitamin D-3 on growth factor secretion in co-cultures of osteoblasts and endothelial cells Ultrasound Med Biol 2000 26 1 161 6 10.1016/S0301-5629(99)00110-6 10687804

[B19] Doan N Reher P Meghji S Harris M In vitro effects of therapeutic ultrasound on cell proliferation, protein synthesis, and cytokine production by human fibroblasts, osteoblasts, and monocytes J Oral Maxillofac Surg 1999 57 4 409 19 10.1016/S0278-2391(99)90281-1 10199493

[B20] Hayton MJ Dillon JP Glynn D Curran JM Gallagher JA Buckley KA Involvement of adenosine 5′-triphosphate in ultrasound-induced fracture repair Ultrasound Med Biol 2005 31 8 1131 8 10.1016/j.ultrasmedbio.2005.04.017 16085103

[B21] Suzuki A Takayama T Suzuki N Sato M Fukuda T Ito K Daily low-intensity pulsed ultrasound-mediated osteogenic differentiation in rat osteoblasts Acta Biochimica Et Biophysica Sinica 2009 41 2 108 15 10.1093/abbs/gmn012 19204827

[B22] Kang KS Lee SJ Lee HS Moon W Cho DW Effects of combined mechanical stimulation on the proliferation and differentiation of pre-osteoblasts Exp Mol Med 2011 43 6 367 73 10.3858/emm.2011.43.6.040 21532314 PMC3128915

[B23] Reher P Elbeshir ENI Harvey W Meghji S Harris M The stimulation of bone formation in vitro by therapeutic ultrasound Ultrasound Med Biol 1997 23 8 1251 8 10.1016/S0301-5629(97)00031-8 9372573

[B24] Li JGR Chang WHS Lin JCA Sun JS Optimum intensities of ultrasound for PGE(2) secretion and growth of osteoblasts Ultrasound Med Biol 2002 28 5 683 90 10.1016/S0301-5629(02)00485-4 12079705

[B25] Park H Yip MC Chertok B Kost J Kobler JB Langer R Zeitels SM Indirect low-intensity ultrasonic stimulation for tissue engineering J Tissue Eng 2010 2010 973530 21350648 10.4061/2010/973530 PMC3039491

[B26] Reher P Harris M Whiteman M Hai HK Meghji S Ultrasound stimulates nitric oxide and prostaglandin E2 production by human osteoblasts Bone 2002 31 1 236 10.1016/S8756-3282(02)00789-5 12110440

[B27] Parvizi J Parpura V Greenleaf JF Bolander ME Calcium signaling is required for ultrasound-stimulated aggrecan synthesis by rat chondrocytes J Orthop Res 2002 20 1 51 10.1016/S0736-0266(01)00069-9 11853090

[B28] Hillam RA Skerry TM Inhibition of bone-resorption and stimulation of formation by mechanical loading of the modeling rat ulna in vivo J Bone Miner Res 1995 10 5 683 9 7639102 10.1002/jbmr.5650100503

[B29] Bikle DD Halloran BP The response of bone to unloading J Bone Miner Metab 1999 17 4 233 44 10.1007/s007740050090 10575587

[B30] Kapur S Baylink DJ Lau KHW Fluid flow shear stress stimulates human osteoblast proliferation and differentiation through multiple interacting and competing signal transduction pathways Bone 2003 32 3 241 51 10.1016/S8756-3282(02)00979-1 12667551

[B31] Dyson M Nonthermal cellular effects of ultrasound Br J Cancer 1982 45 165 71 PMC2149297 6950755

[B32] Tsai CL Chang WH Liu TK Preliminary studies of duration and intensity of ultrasonic treatments on fracture repair Chin J Physiol 1992 35 1 21 6 1424952

[B33] Robling AG Hinant FM Burr DB Turner CH Shorter, more frequent mechanical loading sessions enhance bone mass Med Sci Sports Exerc 2002 34 2 196 202 10.1097/00005768-200202000-00003 11828225

[B34] Angle SR Sena K Sumner DR Virdi AS Osteogenic differentiation of rat bone marrow stromal cells by various intensities of low-intensity pulsed ultrasound Ultrasonics 2011 51 3 281 8 10.1016/j.ultras.2010.09.004 20965537

[B35] Appleford MR Oh S Cole JA Protivínský J Ong JL Ultrasound effect on osteoblast precursor cells in trabecular calcium phosphate scaffolds Biomaterials 2007 28 32 4788 94 10.1016/j.biomaterials.2007.06.010 17706764 PMC2001312

[B36] de GusmÃ£o C Pauli J Saad M Alves J Belangero W Low-intensity ultrasound increases FAK, ERK-1/2, and IRS-1 expression of intact rat bones in a noncumulative manner Clin Orthop Relat Res 2010 468 4 1149 56 10.1007/s11999-009-1146-6 19851814 PMC2835591

[B37] Watabe H Furuhama T Tani-Ishii N Mikuni-Takagaki Y Mechanotransduction activates α5β1 integrin and PI3K/Akt signaling pathways in mandibular osteoblasts Exp Cell Res 2011 317 18 2642 9 10.1016/j.yexcr.2011.07.015 21824471

[B38] Burnstock G Verkhratsky A Long-term (trophic) purinergic signalling: purinoceptors control cell proliferation, differentiation and death Cell Death Dis 2010 1 1 10 10.1038/cddis.2009.2 PMC3032501 21364628

[B39] Genetos DC Geist DJ Liu D Donahue HJ Duncan RL Fluid shear-induced ATP secretion mediates prostaglandin release in MC3T3-E1 osteoblasts J Bone Miner Res 2005 20 1 41 9 10.1359/JBMR.041009 15619668 PMC2929123

[B40] Gardinier JD Majumdar S Duncan RL Wang L Cyclic hydraulic pressure and fluid flow differentially modulate cytoskeleton re-organization in MC3T3 osteoblasts Cell Mol Bioeng 2009 2 1 133 43 10.1007/s12195-008-0038-2 20161062 PMC2747752

[B41] Li J Liu D Ke HZ Duncan RL Turner CH The P2X7 nucleotide receptor mediates skeletal mechanotransduction J Biol Chem 2005 280 52 42952 9 10.1074/jbc.M506415200 16269410

[B42] Kremkau FW Diagnostic Ultrasound: Principles and Instruments 2006 7 Philadelphia: W.B. Saunders Elsevier

